# CircECE1 activates energy metabolism in osteosarcoma by stabilizing c-Myc

**DOI:** 10.1186/s12943-020-01269-4

**Published:** 2020-10-26

**Authors:** Shuying Shen, Teng Yao, Yining Xu, Deguang Zhang, Shunwu Fan, Jianjun Ma

**Affiliations:** 1 Department of Orthopaedic Surgery, Sir Run Run Shaw Hospital, Medical College of Zhejiang University & Key Laboratory of Musculoskeletal System Degeneration and Regeneration Translational Research of Zhejiang Province, 3 East Qingchun Road, Hangzhou, 310016 Zhejiang Province China; 2 Department of Head and Neck Surgery, Institute of Micro-Invasive Surgery of Zhejiang University, Sir Run Run Shaw Hospital, Medical College of Zhejiang University, 3 East Qingchun Road, Hangzhou, 310016 Zhejiang Province China

**Keywords:** Osteosarcoma, *CircECE1*, C-Myc, Glucose metabolism, TXNIP

## Abstract

**Background:**

Osteosarcoma (OS) is the most common malignant bone tumor and has a poor prognosis. The potential involvement of circular RNAs (circRNAs) in OS progression remains unexplored.

Here, we report that *CircECE1*, a circular RNA derived from human *ECE1*, plays a critical role in energy metabolism in OS.

**Methods:**

The RIP chip sequence assay was performed to confirm *CircECE1*, through overexpression or knockdown of *CircECE1* to verify its function in 143B and U2OS. RNA immunoprecipitation and immunoprecipitation were used to verify *CircECE1*’s regulation of protein c-Myc and co- immunoprecipitation was used to verified the competitive binding relationship between *CircECE1* and SPOP. The influence of *CircECE1* on energy metabolism was evaluated by seahorse experiment, western blot, and immunohistochemistry.

**Results:**

We found that *CircECE1* is highly expressed in OS tissues and cells and that *CircECE1* knockdown suppresses tumor proliferation and metastasis both in vitro and in vivo. Further, *CircECE1* significantly promotes glucose metabolism in OS cells in vitro and in vivo. Mechanistically, *CircECE1* interacts with c-Myc to prevent speckle-type POZ-mediated c-Myc ubiquitination and degradation. C-Myc inhibits thioredoxin binding protein (*TXNIP)* transcription and subsequently activates the Warburg effect.

**Conclusions:**

*CircECE1* regulates the Warburg effect through the c-Myc/TXNIP axis. CircECE1 mediated signal transduction plays a important role in OS process and energy metabolism. These findings may identify novel targets for OS molecular therapy.

**Supplementary Information:**

The online version contains supplementary material available at 10.1186/s12943-020-01269-4.

## Background

Although osteosarcoma (OS) is a rare malignancy, it has the second highest incidence and mortality among malignant bone tumors. OS is most common during childhood and adolescence [1, 2]. It is characterized by direct formation of osteoid tissue and uncontrolled proliferation of bone-related mesenchymal cells and is highly aggressive; 75% of OS cases have invasion of nearby tissues [2, 3]. Although neoadjuvant therapy and wide tumor excision have improved survival, the clinical outcomes and survival rates of OS patients are still unfavorable due to early lung-targeted metastasis. Hence, a better understanding of the biological characteristics and molecular mechanisms of OS carcinogenesis is urgently needed.

With the development of RNA high-throughput sequencing technology and advances in biotechnology, many noncoding RNAs have been found to perform a variety of biological functions in the human body and to participate in the occurrence and development of tumors and other diseases. Circular RNAs (circRNAs), an enigmatic subclass of endogenous long noncoding RNAs that regulate genes at the transcriptional or posttranscriptional level, are found in many types of cancer [4–10]. As opposed to linear RNA, circRNA is formed by pre-miRNA through back-splicing of covalently joined 3′- and 5′-ends without the polyadenylated tail. Due to their closed loop structure, circRNAs are able to escape degradation by exonucleases and are much more stable than linear RNAs. Studies have shown that circRNAs serve multiple functions, including acting as miRNA sponges, regulating gene transcription, adsorbing RNA-binding proteins, and regulating protein translation. ^(8,9)^ CircRNAs have diverse functions and mechanisms of biogenesis and have been implicated in multiple cancer types.^10^ Our previous study showed that circRNA modulates OS progression [11–13]; however, further research is needed to clarify the functions and roles of circRNA in OS.

Aerobic glycolysis, also termed the Warburg effect, is the most distinguishing difference between normal cells and malignant tumor cells [14–17]. Aerobic glycolysis refers to the preference for glycolysis over oxidative phosphorylation even when oxygen is sufficient. Tumor cells mainly employ the glycolytic pathway to provide energy, and glucose is primarily processed into lactate. Although the efficiency of aerobic glycolysis is unfavorable for energy generation, the ability of tumor cells to evade apoptosis, invade, perform biosynthesis, and resist chemotherapeutics is significantly improved. Additionally, the preference for aerobic glycolysis over oxidative phosphorylation allows cancer cells to adapt and survive when exposed to hypoxic and acidic environments [18–20]. Emerging evidence indicates that aberrant metabolism is one of the hallmarks of malignant tumors. The molecular mechanisms underlying the transformation of metabolism are related to activation of oncogenes or loss of tumor suppressors, which ultimately lead to stabilization of hypoxia-inducible factor (HIF) 1α or increased expression of the c-Myc oncogene. The transcription factors HIF1α and c-Myc promote the expression of glycolytic genes, thus enhancing lactate production and glycolysis [21–23].

Most current circRNA studies focus on the function of circRNAs as endogenous competitive RNAs. Here, we provide the first evidence for a molecular mechanism by which circRNAs enhance the Warburg effect through binding to c-Myc. The results of the current study reveal a new mechanism of osteosarcoma development and demonstrate the significance of the Warburg effect in osteosarcoma.

## Methods

### RIP-array assay

A RIP experiment was used to extract circRNA bound to c-Myc in 143B cells. After the samples were qualified, a Total RNA-seq (H/M/R) Library Prep Kit for Illumina® was used to construct the library. First, the designed DNA probe was hybridized with the RNA sample, thereby removing rRNA from the total RNA. Fragmented RNA was used to synthesize the first strand of cDNA. Using a strand-specific method, dUTP was incorporated during synthesis of the second strand of cDNA to label it, and at the same time, end repair was completed in this step. Then, A-tails and ligation adapter were added, ligation products were purified, fragments were sorted according to size, the library was amplified, and the second strand template with dUTP was digested with UDG enzyme before PCR amplification. After amplification, the RIP-Seq library was obtained by purification and recovery with magnetic beads. After the library was constructed, Qubit3.0 was used for preliminary quantification, and then, an Agilent2100 Bioanalyzer was used to detect the size range of the library. When the inserted target fragment size met expectations, Q-PCR was performed to accurately quantify the effective concentration of the library (Library effective concentration > 3 nM) to ensure the quality of the library. Finally, the circRNA sequences were tested with a Novaseq6000 system using a paired-end 150-bp reading strategy (PE150 mode). The amount of sequencing data was 10G.

#### Cell culture

The human cell line hFOB1.19 and the human osteosarcoma cell lines MG-63, U2OS, SJSA-1, HOS, and 143B were purchased from FuHeng Cell Center (Shanghai, China). The OS cell lines were authenticated at ShangHai Biowing Applied Biotechnology Co. Ltd. by performing an STR profiling analysis as described by Capes-Davis and according to the ANSI Standard (ASN-0002) set forth by the ATCC Standards Development Organization. Mycoplasma testing was performed using a Venor GeM Mycoplasma Detection Kit (Minerva Biolabs, Berlin, Germany).

#### Ethics

All animal experiments were performed according to the Guide for the Care and Use of Laboratory Animals published by the National Institutes of Health and were approved by the Ethics Committee of Sir Run Run Shaw Hospital. All experiments strictly followed the specific guidelines of the panel regarding the care, treatment, and euthanasia of animals used in the study.

#### Clinical samples

Slices of formalin-fixed and paraffin-embedded primary osteosarcoma and chondroma tissues were obtained from biopsies of each patient prior to administration of neo-adjuvant chemotherapy. Osteosarcoma and chondroma biopsies were histologically characterized by pathologists according to the criteria defined by the World Health Organization. Written informed consent was obtained from each patient prior to study participation, and all study protocols were approved by the Ethics Committee of Sir Run Run Shaw Hospital.

#### Xenograft tumorigenesis

Nude mice (4–6 weeks old) were subcutaneously administered 5 × 10^6^ stable 143B cells suspended in PBS on one side of the lower dorsal flank (*n* = 6 per group). The width and length of the resulting tumors were measured every week for up to 4 weeks, and tumor volume was calculated according to the following formula: volume (mm3) = (length × width2)/2. At 4 weeks postinjection, mice were sacrificed, and tumors were harvested and weighed. Tumor tissues were then used for protein extraction and RNA extraction or fixed for IHC staining.

#### Tail vein metastasis and bioluminescence imaging

Cells (143B) were stably transfected with Luc-vector, Luc-circECE1 WT, or Luc- circECE1 MUT. Mice (4–6 weeks old) were then injected with 1 × 10^6^ stable cells via the tail vein. After 4 weeks, mice were anesthetized using isoflurane and intraperitoneally injected with 150 mg/kg d-luciferin (Yeason, Shanghai, China). After 15 min, tumors exhibiting luciferase expression were imaged using an IVIS Spectrum in vivo imaging system (Xenogen, Caliper Life Sciences). Images were analyzed using Living Image 4.1 software (Xenogen, Caliper Life Sciences).

#### Plasmids

Overexpression vectors for CircECE1 WT or MUT were constructed (BersinBio, Guangzhou, China). The Flag-tagged coding sequences of human c-Myc and TXNIP were cloned into the lentiviral pDC311-U6-MCMV-EGFP vector (purchased from Hanbio Co. Ltd., Shanghai, China) to generate c-Myc and TXNIP expression plasmids.

#### Cell viability

Cell viability was determined daily using a CCK-8 kit (Cell Counting Kit-8; Dalian Meilun Biotechnology Co., Ltd) according to the manufacturer’s instructions. All observations were reproduced at least six times in independent experiments.

#### Anoikis assay

For anoikis analysis, OS cells were cultured in suspension for 48 h. Cells were then trypsinized and stained with Annexin V- APC/7AAD and analyzed by flow cytometry using an Annexin V- APC/7AAD Apoptosis Detection kit (BD Biosciences) according to the manufacturer’s instructions. Data were collected on a BD FACSCan and analyzed using the FlowJo software.

#### Colony formation assay

OS Cells were seeded in triplicate into 6-well plates at an initial density of 500 cells/well. After 14 days, colonies were fixed with 4% paraformaldehyde for 20 min at room temperature (25 °C) and then stained with 5 mg/ml crystal violet (Sigma). Colonies containing more than 50 OS cells were counted using light microscopy. The average number of colonies was determined from three independent experiments.

#### RNA extraction and quantitative real-time PCR analysis

Total cellular RNA was extracted from OS cells or tumor tissues using TRIzol reagent (Invitrogen, Carlsbad, CA, USA) according to the manufacturer’s instructions. RNA was stored at − 80 °C. Reverse transcription was performed using 1.0 μg total RNA and a HiFiScript cDNA Kit (CWBIO, Beijing, China) to investigate the expression of mRNA. Amplification reactions were performed in 20 μl reaction volumes containing amplification primers and UltraSYBR Mixture (with ROX) (CWBIO) that was detected by an ABI 7500 Sequencing Detection System (Applied Biosystems, Foster City, CA, USA). For each amplification reaction, 1 μl cDNA and 1 μl primer (Sangon Biotech, Shanghai, China) were used. The cycling conditions were as follows: 40 cycles of denaturation at 95 °C for 5 s and amplification at 60 °C for 24 s. For circRNA, total RNA was incubated with or without 3 U/μg RNase R (Epicentre, San Diego, CA, USA) at 37 °C for 20 min, and the resulting RNA was subsequently purified using an RNeasy MinElute Cleanup Kit (Qiagen). Specific divergent primers for the back-splice junction of CircECE1 were used to amplify circRNA. The amplified products were detected by agarose gel electrophoresis and sequencing. All reactions were performed in triplicate, and the results were normalized to the mRNA house-keeping gene.

#### Western blotting

Following treatment or transfection, culture supernatants were removed, cells or tissues were lysed using radio immunoprecipitation assay buffer (RIPA, Beyotime, China), and protein was harvested and quantified by bicinchoninic acid (BCA) analysis (Beyotime, China). Protein extracts were separated in 10% SDS-PAGE gels and transferred onto polyvinylidene fluoride (PVDF) membranes (Sigma-Aldrich, USA). After incubation with a high affinity anti-c-Myc antibody (1:1000, Abcam), anti-ELK1 antibody (1:1000, Abcam), anti-cdc25c antibody (1:1000, Abcam), anti-hif1 antibody (1:1000, Abcam), anti-c-Jun antibody (1:1000, Abcam), anti-Mdm2 antibody (1:1000, Abcam), anti-JunB antibody (1:1000, Abcam), anti-mct4 antibody (1:1000, Abcam), anti-pdk1 antibody (1:1000, Abcam), anti-pdk4 antibody (1:1000, Abcam), anti-GLUT1 antibody (1:1000, Abcam), anti-GLUT4 antibody (1:1000, Abcam), anti-Ubiquitin antibody (1:1000, Abcam) or anti-β-actin antibody (1:2000, Cell Signaling Technology, USA) in Primary Antibody Dilution (MB9881, Dalian Meilun Biotechnology Co., Ltd), the membranes were incubated with a secondary antibody (1:5000, Cell Signaling Technology, USA). After washes, signals were detected using FDbio-Femto ECL (Fudebio, Hangzhou, China) and a chemiluminescence system (Bio-Rad, USA). All images were analyzed using Image Lab software.

#### RNA in situ hybridization

Cy3-labeled c-Myc probes and Alexa Fluor 488-labeled circECE1 probes were designed and synthesized by RiboBio (Guangzhou, China), and the probes sequences are available upon request. Probe signals were detected using a Fluorescent InSitu Hybridization Kit (RiboBio, Guangzhou, China) according to the manufacturer’s instructions. The images were acquired on a Nikon A1Si Laser Scanning Confocal Microscope (Nikon Instruments Inc., Japan).

#### Glycolysis analysis

Glucose Uptake Colorimetric Assay Kits (Biovision, USA) and Lactate Colorimetric Assay Kits (Biovision, USA) were used according to the manufacturers’ protocols to examine the glycolysis process in osteosarcoma. A Seahorse XF Cell Mitostress test kit and Bioscience XF96 Extracellular Flux Analyzer were used to measure the oxygen consumption rate (OCR) and extracellular acidification rate (ECAR). Briefly, 4 × 104 OS cells were seeded into 96-well plates and then incubated overnight. After the cells were washed with Seahorse buffer, oligomycin, FCCP, and rotenone were automatically injected to measure the OCR. Then, glucose, oligomycin, and 2-deoxy-glucose (2-DG) were added to measure the ECAR. The OCR and ECAR values were calculated after normalization to the OS cell number and are plotted as the mean ± SD.

#### Measurement of mitochondrial membrane potential

The alteration in the mitochondrial membrane potential (ΔΨm) was measured using a JC-1 kit (Beyotime Biotechnologies, Jiangsu, China). Briefly, U2OS and 143B cells were harvested by scraping and then resuspended in 0.5 mL of culture medium. Next, 0.5 mL of JC-1 Staining Solution was added to the cell suspension. Then, the suspension was incubated for 25 min at 37 °C in a CO_2_ incubator. Next, the OS cells were collected by centrifugation at 300×g for 10 min and washed twice with JC-1 Staining Buffer. Subsequently, 500 μL of JC-1 Staining Buffer was added to each tube, and the OS cells were resuspended. The samples were then analyzed by flow cytometry. Mitochondrial depolarization was measured by a downregulation in the red/green fluorescence intensity ratio.

#### Chromatin immunoprecipitation (ChIP) assay

ChIP assays were performed using an EZ-ChIP kit from Millipore according to the manufacturer’s protocol. The primers used to detect TXNIP promoter occupancy were F: 5′-CAGAGCGCAACAACCATT-3′ and R: 5′-AGGCTCGTGCTGCCCTCGTGCAC-3′.

#### Statistical analysis

Statistical analyses were performed using 22.0 SPSS software. The data are presented as the means with SDs, and statistical significance was determined using unpaired Student’s t-tests. The data were analyzed using paired or unpaired t-tests or ANOVA, as appropriate. A *P*-value of < 0.05 was considered to be statistically significant.

## Results

### CircECE1 is overexpressed in OS tissues and cell lines and is predominantly localized in the cytoplasm

**Fig. 1:**
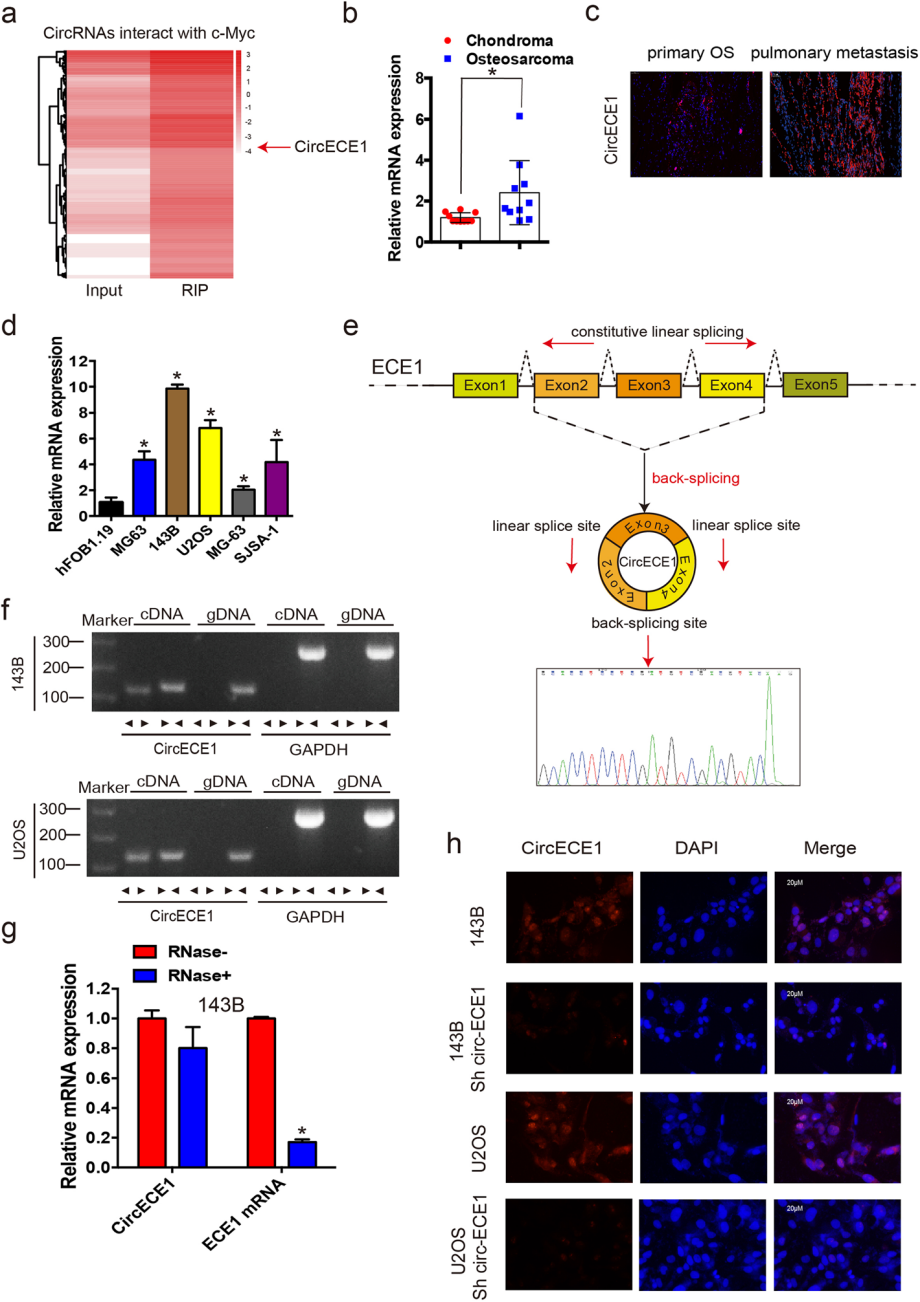
CircECE1 validation and expression in osteosarcoma tissue and cells. **a**. RIP-seq results indicate that CircRNAs interact with c-Myc. The red arrow indicates CircECE1. **b**. Total RNAs were isolated from the specimens of patients with OS and from chondroma tissues for use in real-time PCR. Tumor samples exhibited significantly higher levels of CircECE1 compared to those in the chondroma tissue. Data represent the mean ± SD (*n* = 10). **c**. CircECE1 expression was higher in human OS pulmonary metastasis than in primary OS tissues. Representative images are provided (400× magnification). **d**. CircECE1 expression in hFOB1.19 and osteosarcoma (OS) cell lines (OS-732, 143B, HOS, SJSA-1, and MG-63) was evaluated by qRT-PCR. Data represent the mean ± standard deviation (SD) (*n* = 3). * *P* < 0.05. **e**. Schematic illustration reveals the ECE1 exon 2–4 circularization that forms CircECE1 (red arrow). The presence of CircECE1 was validated by RT–PCR and subsequent Sanger sequencing. The red arrow represents “head-to-tail” CircECE1 splicing sites. **f**. The presence of CircECE1 was validated in U2OS and 143B osteosarcoma cell lines by RT–PCR. Divergent primers amplified circECE1 in cDNA but not in genomic DNA. GAPDH was used as a negative control. **g**. The expression of CircECE1 and ECE1 mRNA in 143B cells treated with or without RNase R was detected by real-time PCR. The relative levels of CircECE1 and ECE1 mRNA were normalized to the value measured in the mock treatment. Data represent the mean ± SD (n = 3). * *P* < 0.05. **h**. RNA fluorescence in situ hybridization (FISH) revealed that CircECE1 was predominantly localized within the cytoplasm. CircECE1 probes were labeled with Alexa Fluor 488, and nuclei were stained with DAPI. Scale bar = 50 μm

The RNA immunoprecipitation (RIP) microarray expression profile for detecting circRNAs binding c-Myc in OS cell lines is described in Fig. 1a. We chose 10 circRNAs (input > 0.2, fold change > 10, and fdr < 0.05) to verify binding to c-Myc in 143B cells and found that *hsa_circ_0002402*, or *CircECE1*, was most significantly enriched (up to 25.13-fold, Supplementary Fig. 1). To investigate the expression of *CircECE1* in clinical samples, we collected 10 chondroma and 10 OS samples and performed qRT-PCR to detect *CircECE1* expression. *CircECE1* was more highly expressed in OS tissue than in chondroma tissue (Fig. 1b). Further, the expression of *CircECE1* was higher in pulmonary metastases than in primary OS tissues (Fig. 1c). The expression of *CircECE1* was also higher in OS cell lines (HOS, 143B, U2OS, SJSA-1, and MG-63) than in the hFOB1.19 cell line. Among the OS cell lines, U2OS and 143B cells had the highest expression of *CircECE1* (Fig. 1d). *CircECE1* is generated from the *ECE1* gene located on chromosome 1. The annotation for *circECE1* includes exons 2, 3, and 4 of *ECE1* (total 442 bp) (Fig. 1e). Therefore, we assessed the head-to-tail splicing of endogenous *CircECE1* by RT-PCR with convergent and divergent primers. The divergent primers for *CircECE1,* but not *GAPDH,* amplified a PCR product, indicating a circular and not linear form (Fig. 1f). Similarly, qPCR analysis of total RNA after RNase R treatment indicated that *CircECE1* was resistant, whereas *ECE1* mRNA transcripts decreased sharply under RNase R treatment (Fig. 1g). RNA FISH revealed that *CircECE1* was mainly localized in the cytoplasm and nucleus (Fig. 1). Taken together, these findings reveal that *CircECE1* is overexpressed in OS tissues and cell lines and is localized in the cytoplasm and nucleus.

### CircECE1 promotes the migration and proliferation of OS cells

**Fig. 2:**
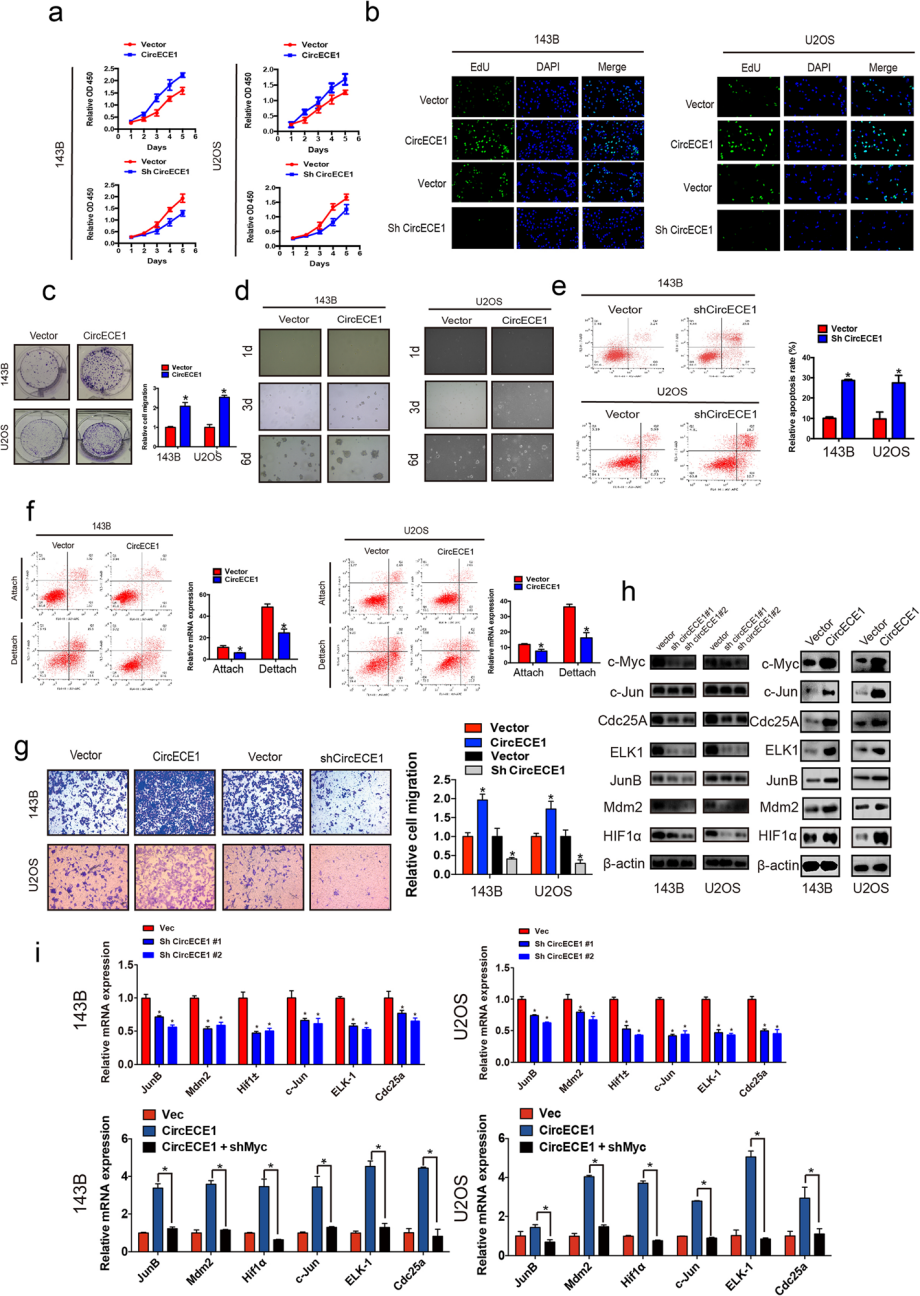
CircECE1 increased cell survival and colony formation and promoted the expression of c-Myc targets. **a**. Silencing or overexpression of CircECE1 in osteosarcoma cells decreased or increased, respectively, the ability of cell proliferation compared to that of the control. **P < 0.01. Data represent the mean ± SD (*n* = 6). **b**. CircECE1 functions in tumor cell proliferation as detected by EdU assay. Nuclei were stained with DAPI, and a combined reaction involving EdU and DAPI indicated the cells in S phase. **c**. CircECE1 overexpression promotes cell growth as determined by colony formation assay (details are shown in the insets). Error bars represent the mean ± SD of three independent experiments. * P < 0.05. **d**. Cellular transformation was induced by CircECE1 overexpression. Vector or CircECE1-overexpressing OS stable cells were cultured in soft agar for 20 days. Colonies were stained with crystal violet, photographed, and quantified using ImageJ (details are shown in inserts). **e**. CircECE1 downregulation in osteosarcoma cells increased cell apoptosis (n = 3). **f**. CircECE1 overexpression in osteosarcoma cells decreased cell anoikis (n = 3). **g**. Cell migration abilities of U2OS and 143B cells transfected with CircECE1 or vector were evaluated by transwell migration assays. Data represent the mean ± SD (n = 3). * *P* < 0.05. Scale bar, 50 μm. **h**. Protein lysates from vector and CircECE1 or shCircECE1-transfected osteosarcoma cells were subject to western blotting. Cell lysates were analyzed using c-Myc, HIF-1α, Cdc25a, Mdm2, ELK1, JUN, and JUNB antibodies. **i**. The shCircECE1 or CircECE1 and the vector transfected osteosarcoma cells were maintained at 80% confluence and were used for RNA extraction and measurement of c-Myc and c-Myc targets. The CircECE1 cells expressed significantly higher levels of c-Myc targets compared to those of the control

We designed three CircECE1 siRNAs that specifically targeted the junction sites of CircECE1, transfected U2OS and 143B cells with these siRNAs, and then assessed the knockdown efficiency by qRT-PCR. Knockdown experiments with independent small siRNAs designed against back-splicing between exons 2 and 4 of CircECE1 (Fig. S2a) confirmed that these siRNAs selectively targeted CircECE1, and siRNA#2 provided the most effective knockdown. We then constructed CircECE1 small hairpin RNA (shRNA) using the sequence of siRNA#2 to stably knockdown the expression of CircECE1 in OS cells (Fig. S2b). We also overexpressed CircECE1 and found that it did not affect ECE1 mRNA levels (Fig. S2c). Cell viability was further evaluated with CCK-8 and EDU assays, which indicated that CircECE1 knockdown impaired proliferation and CircECE1 overexpression promoted proliferation (Fig. 2a-b). Overexpression of CircECE1 also significantly promoted the colony-forming ability of OS cells (Fig. 2c). Soft agar assay results also showed that CircECE1 overexpression increased colony formation (Fig. 2d). Moreover, CircECE1 knockdown augmented the apoptosis rate (Fig. 2e), and anoikis in OS cells could be rescued by CircECE1 overexpression (Fig. 2f). The migration ability of OS cells was prominently decreased by CircECE1 shRNA and increased by CircECE1 overexpression (Fig. 2g). Taken together, these findings reveal the role of CircECE1 in the motility, survival, and proliferation of OS cells in vitro.

### CircECE1 regulates c-Myc function. C-Myc regulate the expression of many different genes involved in tumorigenesis

To determine how CircECE1 affects cancer cell behavior, we determined whether CircECE1 could affect c-Myc function target genes. We conducted RT-qPCR and WB and analyzed the expression of some known c-Myc targets (JunB, Mdm2, HIF1α, c-Jun, ELK-1, and Cdc25a) in our CircECE1-overexpressing and knockdown cells and found that several c-Myc targets were upregulated in the CircECE1-overexpressing OS cells and downregulated in CircECE1 knockdown OS cells (Fig. 2h and i). To validate the effect of CircECE1 in mediating c-Myc function, we investigated whether c-Myc knockdown could reverse the changes in target genes induced by CircECE1 overexpression. c-Myc knockdown blocked the upregulation of JunB, Mdm2, HIF1α, c-Jun, ELK-1, and Cdc25a that was induced by CircECE1 overexpression (Fig. 2i). These results indicate that CircECE1 regulates c-Myc function.

### CircECE1 interacts with c-Myc to prevent its degradation by speckle-type POZ (SPOP)

**Fig. 3:**
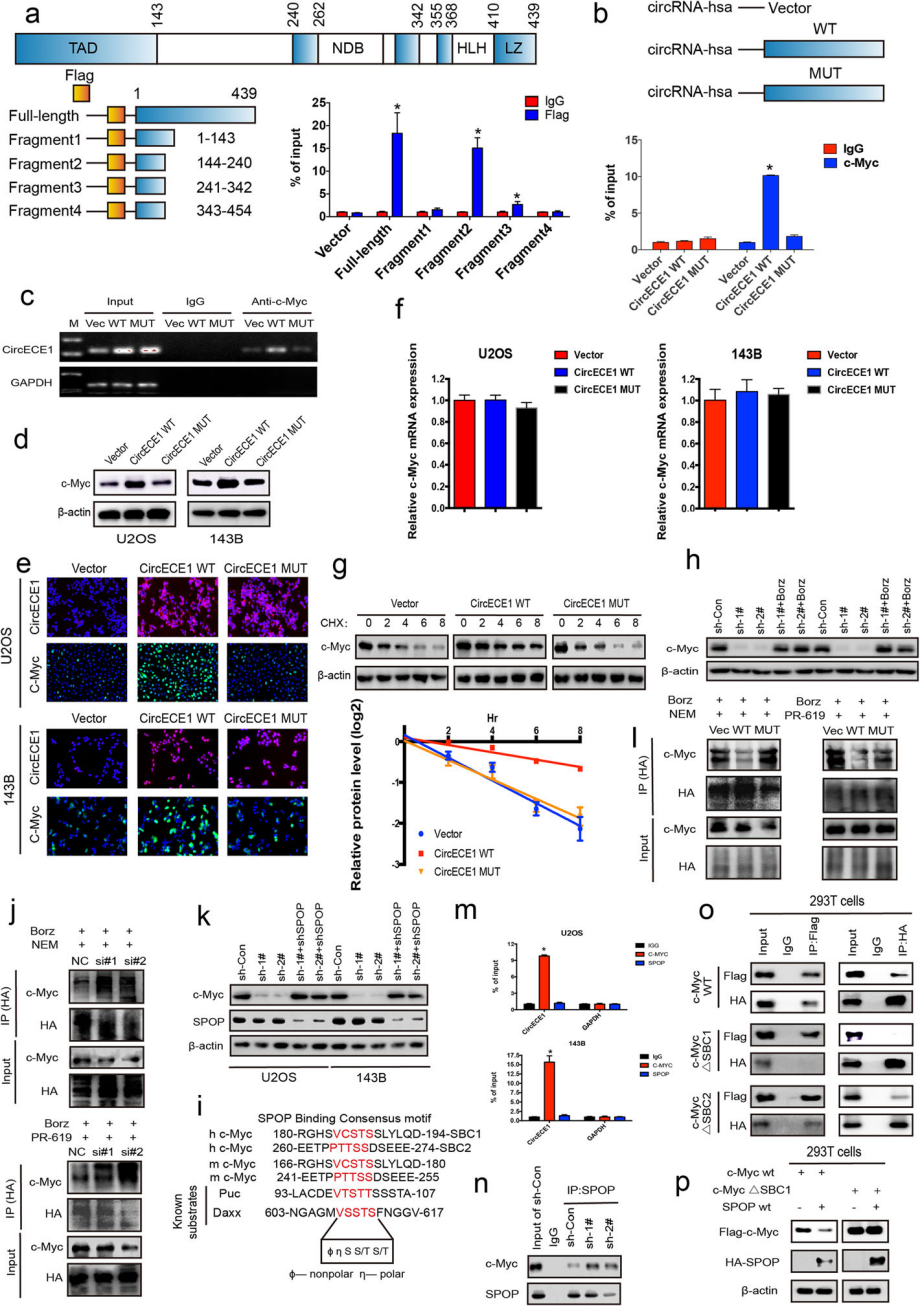
CircECE1 interacts with c-Myc to prevent SPOP-mediated degradation. **a**. Identification of the regions of the c-Myc protein that interact with CircECE1. The fragments of the c-Myc protein are illustrated (left); the interaction of c-Myc protein regions with CircECE1 in 293 T cells was confirmed using a RIP assay (right). The interaction of CircECE1 with c-Myc and SPOP was verified using a RIP assay. **b**-**c**. Schematic diagram of CircECE1 WT and MUT (left); RNA immunoprecipitation experiments were performed using anti-c-Myc antibodies in 143B cells by qRT-PCR (**b**) and RT-PCR(**c**), and specific primers were used to detect CircECE1 or GAPDH; *n* = 3. **d**-**e**. The effect of CircECE1 WT/MUT on the expression of c-Myc protein levels in OS cells as assessed by WB (**d**) and IF (**e**). **f**. The effect of CircECE1 WT/MUT on the expression of c-Myc mRNA levels in OS cells as assessed by qRT-PCR. **g**. The effect of CHX treatment on the change in the c-Myc protein level mediated by CircECE1 WT and MUT overexpression as detected by western blotting. **h**. The effect of Bortezomib treatment on the change in the c-Myc protein level mediated by CircECE1 knockdown as detected by western blotting. **i**. The ubiquitination levels of c-Myc in CircECE1 WT or MUT cells as measured by IP experiments. Bortezomib (250 nM) and NEM (5 μm)(Protease inhibitor) or PR-619 (100 nm)(Non-selective deubiquitinating enzyme inhibitor) were added for 6 h. **j**. The ubiquitination levels of c-Myc in CircECE1 knockdown cells as measured by IP experiments. Bortezomib (250 nM) and NEM (5 μm) or PR-619 (100 nm) were added for 6 h. **k**. The effect of SPOP knockdown on the change in the c-Myc protein level mediated by CircECE1 knockdown as detected by western blotting. **l**. Sequence alignment of c-Myc with the SPOP binding motif (SBC) in known SPOP substrates. **m**. The interaction of CircECE1 WT and MUT with c-Myc in OS cells was verified using a RIP assay. **n**. The interaction of c-Myc with SPOP was verified using a Co-IP assay. **o**. Role of SBC1 and SBC2 in the direct interaction of SPOP with c-Myc. 293 T cells were transfected with expression vectors for SPOP WT and FLAG- c-Myc or with c-Myc that was mutated within the SBC (ΔSBC) 1 or 2 for 24 h. Bortezomib (250 nM) was then added for 6 h. Total cell lysates were prepared, and IP experiment was conducted using anti-FLAG or anti-SPOP antibodies. **p**. 293 T cells were transfected as indicated with HA-SPOP WT and Flag-c-Myc or Flag-c-Myc ΔSBC1 for 24 h. Immunoblot analyses were conducted to detect for Flag-tagged c-Myc, HA-tagged SPOP, and β-actin

We used two bioinformatic websites (catRAPID and CISBP-RNA) to predict the binding sites of CircECE1 and c-Myc (Fig. S3a-b). To identify the c-Myc regions that interact with CircECE1 in vitro, HEK-293 T cells were cotransfected with CircECE1 and vectors encoding various fragments of the c-Myc protein (Fig. 3a). Our results demonstrated that c-Myc fragment 2 interacted with CircECE1 (Fig. 3b), which matched the predicted binding sites. Next, a series of CircECE1 truncations were constructed to map the CircECE1 fragment that interacts with c-Myc (Fig. S3c). RIP results indicated that truncation 2 and 4 of CircECE1 interacts with c-Myc (Appendix Fig. S3D). Furthermore, we mutated four predicted binding sites in the truncation 2 (100–130 bp) and 4 (301–442 bp) region of CircECE1 to determine whether this mutated form still interacted with c-Myc (Fig. S3e). We found that WT CircECE1, but not MUT CircECE1, interacted with c-Myc in 143B cells (Fig. 3b-c & Fig. S3f).

Additionally, we found that *CircECE1* overexpression resulted in increased levels of c-Myc protein in U2OS and 143B cells (Fig. 3d and e) but not increased mRNA levels (Fig. 3f). Given these observations, we hypothesized that *CircECE1* regulates c-Myc levels in OS cells posttranscriptionally. To assess this possibility, *CircECE1*-WT/MUT-overexpressing cells were treated with the protein synthesis inhibitor CHX and *CircECE1* knockdown cells were treated with the proteasome inhibitor Bortezomib. CHX treatment decreased c-Myc protein in 143B cells (Fig. 3g). The half-life of c-Myc was significantly longer in *CircECE1* WT cells than in control or *CircECE1* MUT cells (Fig. 3g). Additionally, Bortezomib treatment reversed the decrease in c-Myc protein induced by *CircECE1* knockdown (Fig. 3h). IP results also indicated that c-Myc was more ubiquitinated in *CircECE1* knockdown cells and less ubiquitinated in *CircECE1*-overexpressing cells compared to control cells after treatment with NEM (Inhibitors of endogenous deubiquitinating enzymes) or PR-619 (Nonselective deubiquitinating enzyme inhibitor) (Fig. 3i-j). These results indicate that *CircECE1* regulates c-Myc levels in OS cells posttranscriptionally.

The E3 ubiquitin ligase adapter SPOP has been reported to promote c-Myc ubiquitination and degradation. To examine whether SPOP is involved in *CircECE1*-mediated changes in c-Myc expression, SPOP was knocked down in *CircECE1* knockdown U2OS and 143B cells. SPOP knockdown abolished the decrease in c-Myc protein levels induced by *CircECE1* knockdown (Fig. 3k). Previous reports have shown that SPOP substrates share an SPOP-binding consensus motif, ϕ π S S/T S/T (ϕ-nonpolar; π-polar). Interestingly, c-Myc has two putative SBC sequences (SBC1: aa 185 VCSTS 189 and SBC2: aa 261 PTTSS 265). Further, SBC1 is located in the *CircECE1* binding site (Fig. 3l). We therefore hypothesized that *CircECE1* may compete with SPOP to block its interaction with c-Myc, thus preventing SPOP-mediated degradation. We first performed a RIP assay, which showed that *CircECE1* interacts with c-Myc, but not SPOP (Fig. 3m). Next, we performed Co-IP experiments, and *CircECE1* knockdown enhanced the interaction between SPOP and c-Myc in 143B cells (Fig. 3n). Notably, while WT c-Myc can bind to the HA-tagged SPOP, c-Myc mutated at SBC1 (ΔSBC1) was unable to bind to SPOP (Fig. 3o). Mutation at SBC2 (ΔSBC2) also impaired the SPOP-c-Myc interaction, but residual binding persisted (Fig. 3p). These data suggest that SBC1 is the major SPOP binding site, whereas SBC2 plays a dispensable role in SPOP-c-Myc binding. All these results indicate that *CircECE1* interacts with c-Myc to prevent SPOP-mediated degradation.

### CircECE1 overexpression promotes the Warburg effect

**Fig. 4:**
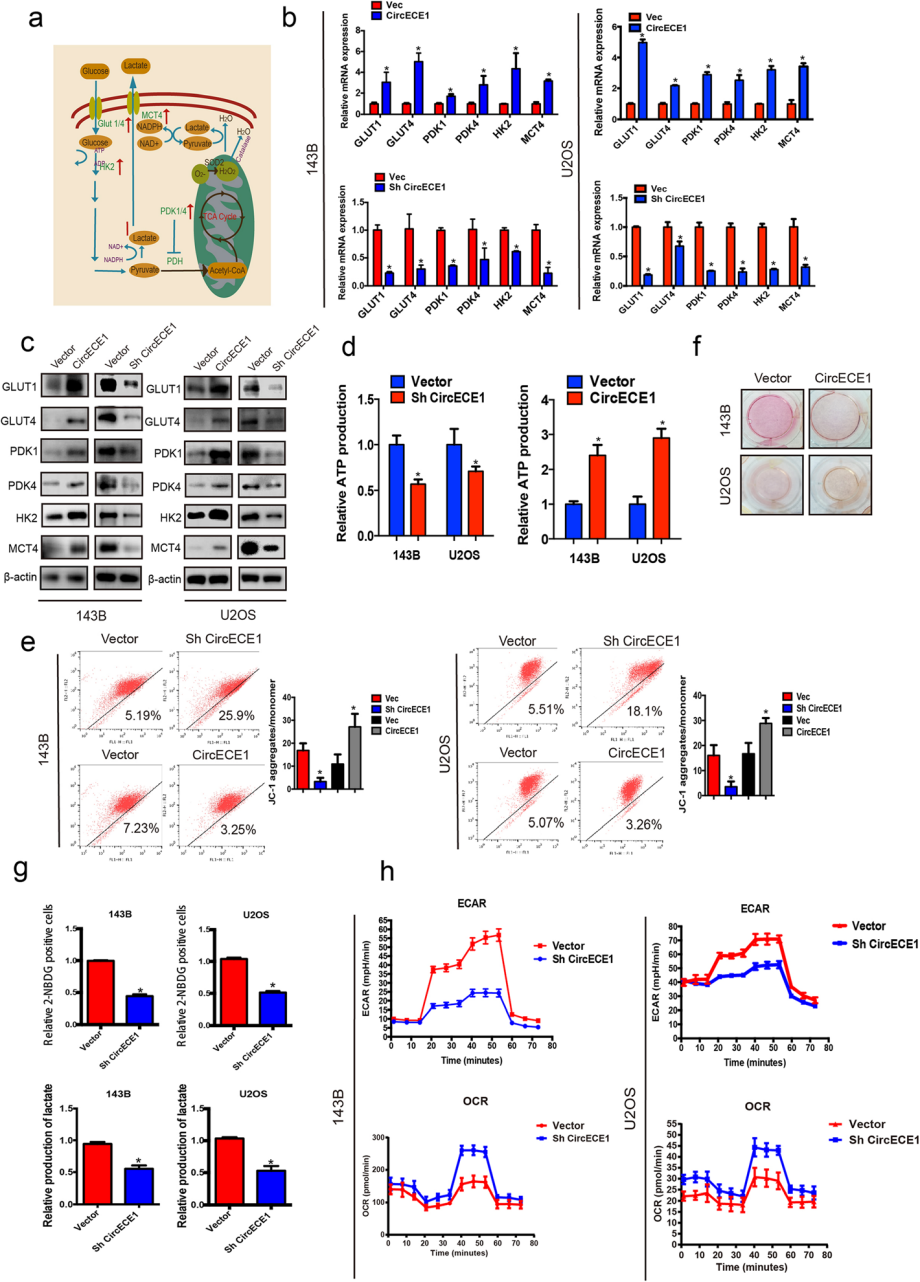
CircECE1 is a positive regulator of glucose metabolism in osteosarcoma. **a**. Schematic representation of glucose metabolism in cancer cells. **b**-**c**. OS cells infected with either control shRNA or CircECE1 shRNA were subjected to various analyses to measure the expression levels of genes involved in glucose metabolism by real-time PCR (**b**) or WB (**c**). OS cells infected with either control vector or CircECE1 overexpression vector were subjected to various analyses to measure the expression levels of genes involved in glucose metabolism by real-time PCR (**b**) or WB (**c**). **d**. CircECE1 overexpression increased ATP production and CircECE1 knockdown decreased ATP production. **e**. Mitochondrial potential increased in the presence of CircECE1 overexpression and decreased in the presence of CircECE1 knockdown. **f**. CircECE1 induced lactate production as indicated by the color of the medium. **g**. CircECE1 downregulation inhibited glucose uptake and lactate production in osteosarcoma cells. **h**. Extracellular acidification rate (ECAR), an indicator of glycolysis, was reduced in response to CircECE1 knockdown. Oxygen consumption rate (OCR), which reflects mitochondrial respiration, was decreased in CircECE1 inhibited osteosarcoma cells

It has been well documented that c-Myc promotes glycolysis through upregulation of various target genes. Therefore, we investigated whether CircECE1 is involved in glycolysis and energy metabolism. To further study the potential function of CircECE1 in the regulation of glucose metabolism, we examined whether CircECE1 overexpression or knockdown affected the expression of a panel of genes involved in glucose metabolism. qPCR and WB analyses revealed that CircECE1 dysregulation affected the expression levels of various key regulators involved in glucose uptake, glycolysis, and lactate secretion (Fig. 4a-c). Specifically, CircECE1 overexpression increased the levels of several glycolysis genes, including Glut1, Glut4, hexokinase 2, and the lactate transporter MCT4 (Fig. 4b-c). CircECE1 overexpression also increased the expression levels of pyruvate dehydrogenase (PDK) 1 and PDK4 (Fig. 4b-c), whereas CircECE1 knockdown had the opposite effect. To meet the demands of rapid proliferation and metastasis, malignant tumor cells rely on glucose metabolism for ATP production. Therefore, we analyzed the impact of CircECE1 on ATP production. CircECE1 overexpression increased ATP production in U2OS and 143B cells, whereas CircECE1 knockdown decreased ATP production (Fig. 4d). The mitochondrial membrane potential, which is used to evaluate early apoptosis, reflects mitochondrial integrity and varies according to the metabolic state. CircECE1 increased the mitochondrial membrane potential in U2OS and 143B cells (Fig. 4e), indicating that CircECE1 also functions as a positive regulator of mitochondrial glucose metabolism. Correspondingly, CircECE1 knockdown led to decreased glucose uptake and lactate production (Fig. 4f-g). Seahorse analysis also indicated an increased extracellular acidification rate (ECAR) and oxygen consumption rate (OCR) in CircECE1-overexpressing cells (Fig. 4h). Taken together, our results revealed that CircECE1 plays a vital role in glucose metabolism in OS cells.

### C-Myc mediates the functions of CircECE1 in glucose metabolism

**Fig. 5:**
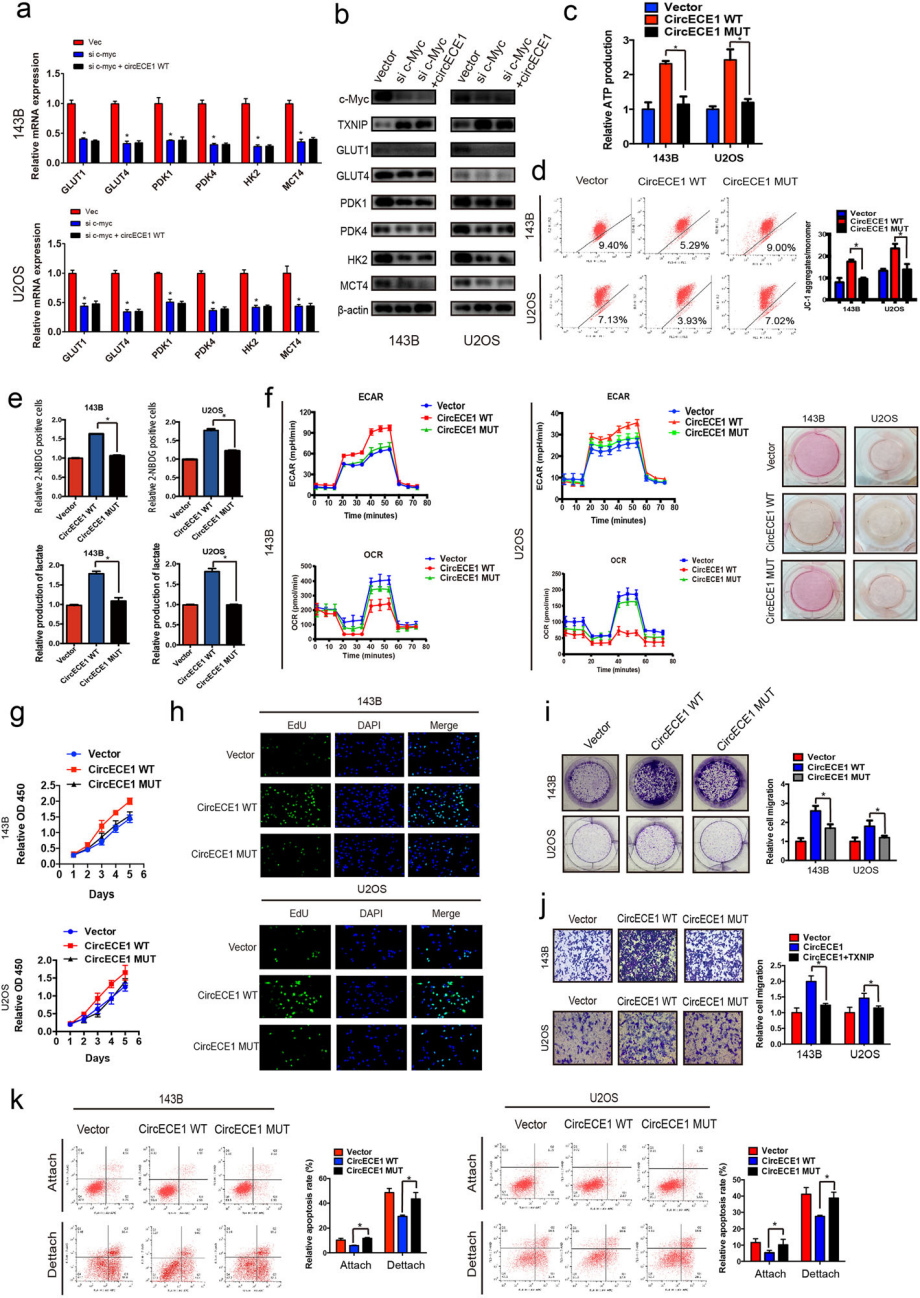
CircECE1 regulates glucose metabolism via c-Myc. **a**-**b**. After knocking down c-Myc, over-expression of circECE1, the protein and RNA of genes related to glucose metabolism were assessed by real-time PCR (**a**) and WB (**b**). **c**. CircECE1 MUT decreased ATP production induced by CircECE1 WT in U2OS and 143B cells. **d**. Mitochondrial potential was decreased after CircECE1 MUT overexpression compared to that of CircECE1 WT. **e**. Glucose uptake and lactate production was lower in the CircECE1 MUT-overexpressing OS cells. **f**. CircECE1 MUT rescued the upregulated the glycolysis rate induced by CircECE1 WT, and this was supported by the ECAR. CircECE1 MUT rescued the upregulated OCR induced by CircECE1 WT. **g**. CircECE1 WT function in tumor cell proliferation as detected by CCK-8 assay. **h**. CircECE1 WT function in tumor cell proliferation as detected by EdU assay. Nuclei were stained with DAPI. The combined reaction between EdU and DAPI indicated cell that were in S phase. **i**. CircECE1 overexpression promoted cell growth based on the results of a colony formation assay (details are shown in the insets). Error bars represent the mean ± SD of three independent experiments. * *P* < 0.05. **j**. Cell migration abilities of U2OS and 143B cells transfected with CircECE1 WT and MUT or vector were evaluated using transwell migration assays. Data represent the mean ± SD (*n* = 3). * *P* < 0.05. Scale bar = 50 μm. **k**. CircECE1 MUT overexpression in osteosarcoma cells increased cell anoikis (*n* = 3) compared to that observed in response to CircECE1 WT

We next investigated whether c-Myc was required for the role of CircECE1 in glucose metabolism. The expression of glycolytic enzymes related to glucose metabolism could not be upregulated after c-Myc was knocked down (Fig. 5a-b). Further, ATP production decreased with CircECE1 MUT overexpression compared to CircECE1 WT overexpression (Fig. 5c). CircECE1 MUT also decreased the mitochondrial membrane potential of U2OS and 143B cells compared to CircECE1 WT (Fig. 5d). Subsequent analysis indicated that CircECE1 MUT inhibited glucose uptake and lactate production compared to CircECE1 WT in U2OS and 143B cells (Fig. 5e). The ECAR and OCR results measured using a Seahorse metabolism analyzer further confirmed that CircECE1 MUT did not have an effect on glycolysis and mitochondrial respiration, but CircECE1 WT did (Fig. 5f). These results confirm that c-Myc mediates the functions of CircECE1 in glucose metabolism.

We then conducted CCK8, EDU, and colony formation assays, which demonstrated that *CircECE1* MUT significantly suppressed the proliferation and colony formation ability of OS cells compared to *CircECE1* WT (Fig. 5g-i). Additionally, the migration abilities of OS cells were prominently decreased by *CircECE1* MUT compared to *CircECE1* WT (Fig. 5j). Moreover, *CircECE1* MUT augmented the apoptosis rate compared to *CircECE1* WT (Fig. 5k). Taken together, these findings indicate that c-Myc mediates the functions of *CircECE1* in glucose metabolism.

### TXNIP is a target of *CircECE1* in OS

**Fig. 6:**
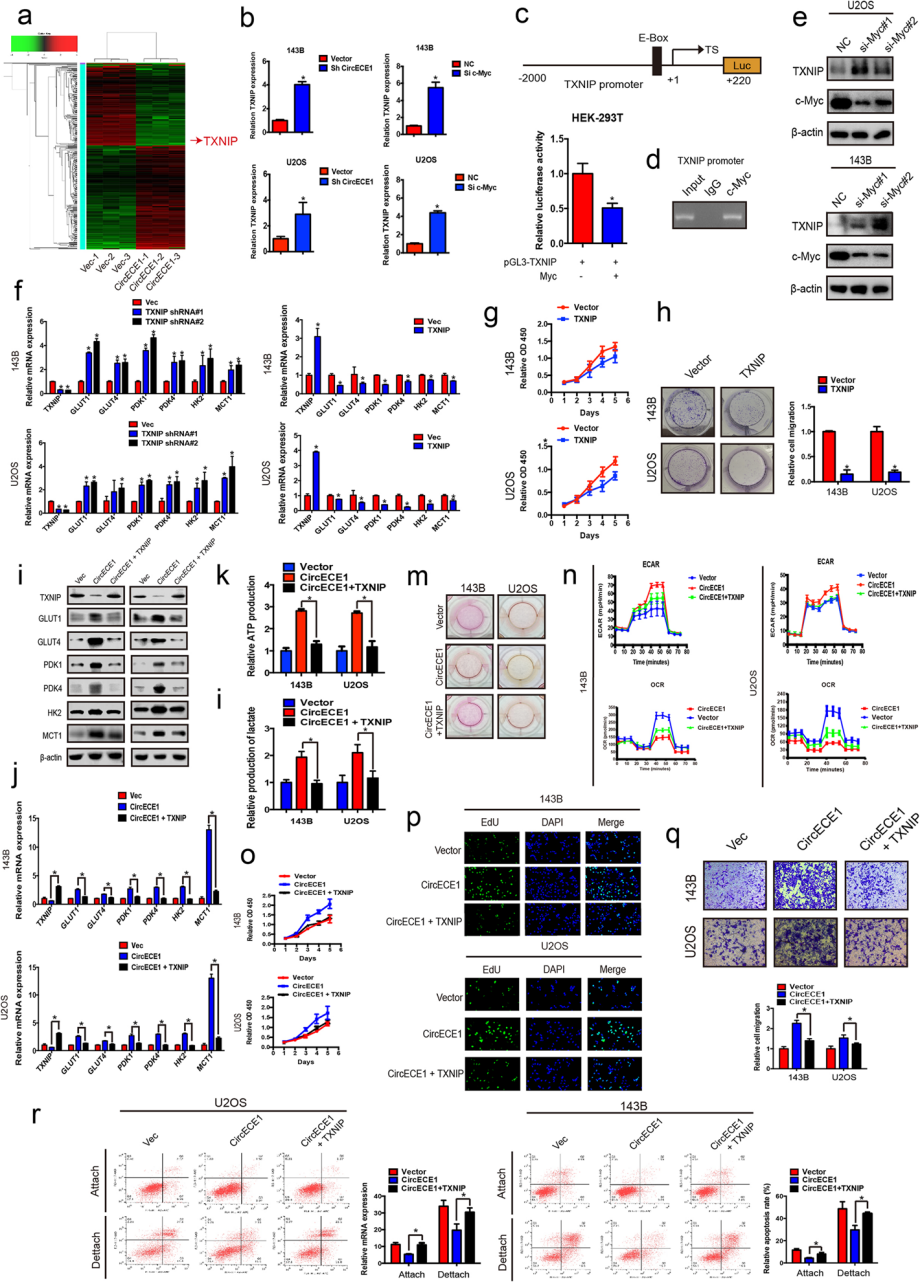
TXNIP negatively regulates glucose metabolism and proliferation in osteosarcoma. **a**. Overexpression of CircECE1 in OS cells, and heat map detailing the RNA-seq. **b**. Knockdown of c-Myc or CircECE1 increased the levels of TXNIP mRNA. **c**. Luciferase assay showed that c-Myc binds to the TXNIP promoter. **d**. c-Myc occupies the E-box of the TXNIP promoter region as measured by ChIP assay. **e**. Knockdown of c-Myc increased TXNIP protein expression. **f**. Effect of TXNIP overexpression or knockdown on the expression levels of genes involved in glucose metabolism in OS cells as assessed by real-time PCR. **g**. TXNIP inhibited cell proliferation as measured by a CCK-8 proliferation kit. **h**. TXNIP inhibited cell proliferation as measured by a colony formation assay. **i**. Cell lysates were collected, and immunoblotting was performed using the indicated antibodies. **j**. TXNIP rescued the expression levels of genes involved in glucose metabolism in OS cells by real-time PCR, and this was induced by CircECE1 overexpression. **k**-**m**. ATP and lactate production was lower in the TXNIP and CircECE1 co-expressing OS cells. **n**. TXNIP and CircECE1 co-expression lowered the glycolysis rate as indicated by the ECAR and OCR. **o**. TXNIP and CircECE1 function in tumor cell proliferation as detected by CCK-8 assay. **p**. TXNIP and CircECE1 function in tumor cell proliferation as detected by EdU assay. Nuclei were stained with DAPI. A combined reaction between EdU and DAPI identified the cells in S phase. **q**. Cell migration abilities of U2OS and 143B cells transfected with CircECE1 and TXNIP, CircECE1 alone, or vector were evaluated using transwell migration assays. Data represent the mean ± SD (n = 3). * *P* < 0.05. Scale bar = 50 μm. **r**. TXNIP and CircECE1 overexpression in osteosarcoma cells increased cell anoikis (*n* = 3) compared to that observed in response to CircECE1 alone

To identify the molecular mechanism underlying the CircECE1-mediated regulation of glucose metabolism, we performed RNA-seq and found a series of signaling pathways altered by CircECE1 overexpression (Fig. 6a). TXNIP, one of the differentially expressed genes, is a well-established regulator of glucose metabolism and is regulated by c-Myc in some cancer cells [24–26]. We first explored whether TXNIP was also regulated by c-Myc in OS and examined its function in OS. Both c-Myc and CircECE1 knockdown significantly altered TXNIP mRNA expression in two OS cell lines (Fig. 6b). Additionally, c-Myc inhibited TXNIP promoter activity in a luciferase assay (Fig. 6c). Moreover, c-Myc occupied the E-boxes in the TXNIP promoter region, as determined by a chromatin immunoprecipitation assay (Fig. 6d), and c-Myc knockdown increased TXNIP protein levels in OS cells (Fig. 6e & Fig. S6a). These findings suggest that c-Myc blocks TXNIP transcription.

We then generated stable U2OS and 143B cell lines with ectopic TXNIP overexpression or with TXNIP knockdown via shRNA (Fig. S6b). A subsequent analysis indicated that TXNIP inhibited the expression of glycolytic enzymes related to glucose metabolism (Fig. 6f). We also examined the influence of TXNIP on OS cell proliferation. As expected, a CCK8 proliferation assay indicated that TXNIP decreased the proliferation of U2OS and 143B cells (Fig. 6g). Colony formation assays demonstrated that TXNIP also inhibited the colony formation ability of OS cells (Fig. 6h).

We hypothesized that TXNIP was a potential effector of *CircECE1* in the regulation of glucose metabolism. To test this hypothesis, we examined the expression of TXNIP in two *CircECE1*-overexpressing OS cell lines. TXNIP protein levels decreased following *CircECE1* overexpression (Fig. 6i). Further, we overexpressed TXNIP in *CircECE1* WT U2OS and 143B cells and found that TXNIP could reverse the effects of *CircECE1* WT overexpression in vitro, including regulating the expression of various key regulators involved in glucose uptake, glycolysis, lactate secretion, ATP production, glucose uptake, lactate production, ECAR, OCR, proliferation, migration, and anoikis (Fig. 6i-r). Therefore, we believe that TXNIP is an important downstream effector of *CircECE1* in regulating glucose metabolism.

### *CircECE1* promotes tumor growth and metastasis and increases glucose utilization in a xenograft model

**Fig. 7:**
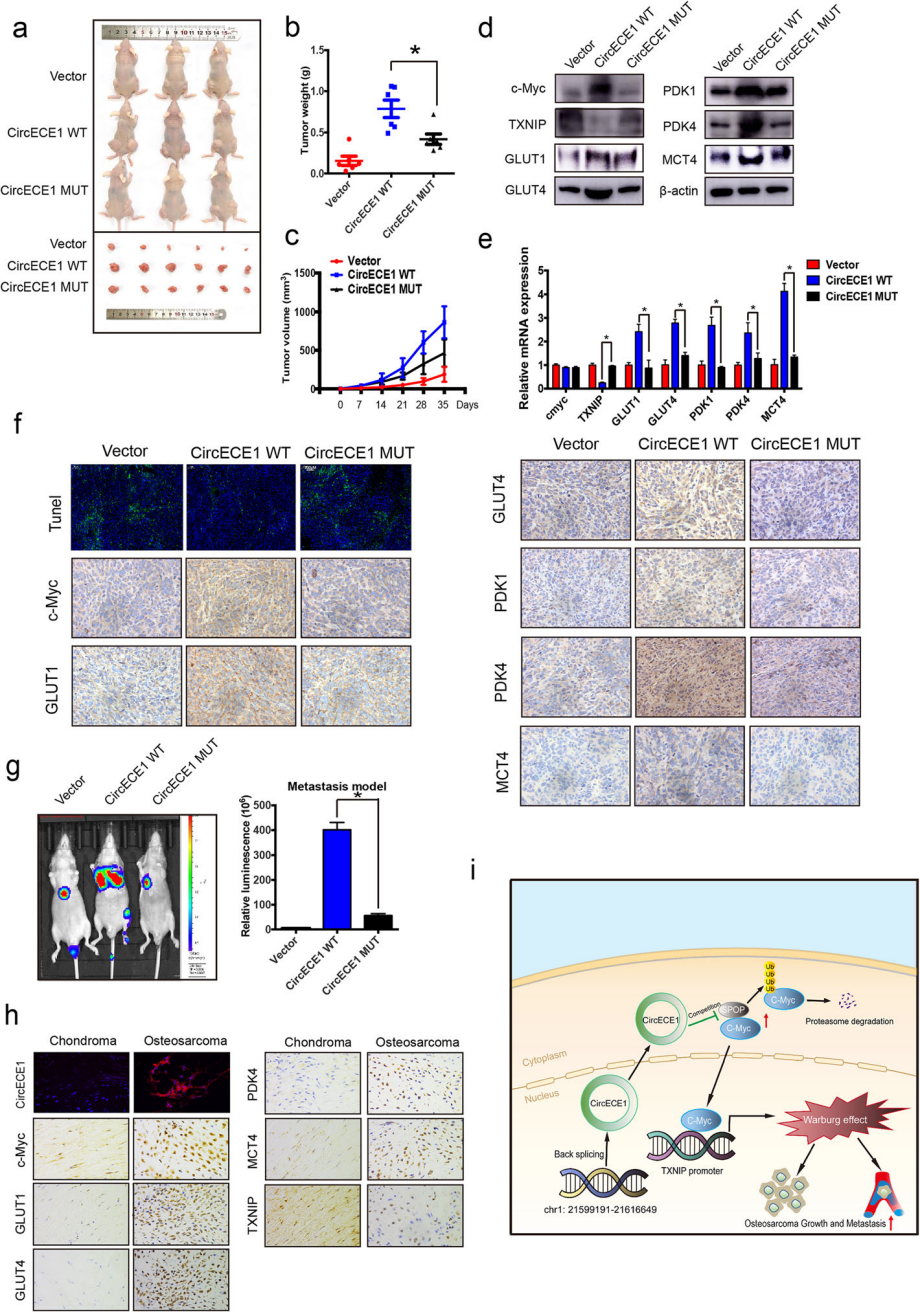
CircECE1 positively regulates glucose metabolism in a xenograft model. **a**. 143B cells stably expressing CircECE1 WT, MUT, or empty vector were subcutaneously injected into nude mice. Nude mice were respectively injected with an equal amount of 5 × 10^6^ stable control cells or cells transfected with CircECE1 WT or MUT subcutaneously. After 30 days, tumors were dissected and photographed. **b**. Tumor weight was calculated on the day mice were euthanized. Data represent the mean ± SEM (*n* = 6 each group). **c**. Tumor volumes (ab2/2) were recorded every six days beginning on the day after mice were injected with the stable OS cells. Data represent the mean ± SEM (n = 6 each group). **d**. Western blotting was used to assess protein expression levels. Expression of the rate-limiting enzymes GLUT1, GLUT4, MCT4, PDK1, and PDK4 was decreased in tumors formed by CircECE1 overexpression in osteosarcoma cells. **e**. RT-qPCR revealed that the mRNA levels of the rate-limiting enzymes GLUT1, GLUT4, MCT4, PDK1, and PDK4 were decreased in tumors formed by CircECE1 overexpression in osteosarcoma cells. **f**. H&E staining and immunohistochemistry (IHC) revealed the structure of OS in mice and the relative protein levels of GLUT1, GLUT4, MCT4, PDK1, and PDK4 in tumors of different groups. Scale bars = 100 μm. **g**. Lung metastasis in mice injected with various stable 143B cells in the tail vein was detected using an in vivo bioluminescence imaging system. Representative images and a histogram are shown (*n* = 9 each group). **h**. Fish revealed the expression level of CircECE1 and Immunohistochemistry (IHC) revealed the relative protein levels of GLUT1, GLUT4, MCT4, PDK1, and PDK4 in chondroma and osteosarcoma. Scale bars = 100 μm. **I**. Schematic illustration of the CircECE1/c-Myc/TXNIP axis

To further confirm the role of CircECE1 in glucose metabolism, we subcutaneously injected nude mice with CircECE1 WT- or MUT-overexpressing 143B cells. As expected, compared with the control or CircECE1 MUT cells, CircECE1 WT cells had a higher tumor growth rate (Fig.7a). We observed similar results for the average tumor wet weight and tumor volume in the three groups (Fig. 7b-c). Subsequent WB, RT-qPCR, and immunohistochemistry analysis using antibodies against GLUT1, GLUT4, PDK1/4, and MCT4 indicated that the mRNA and protein levels of these glycolytic enzymes were significantly upregulated in tissues from CircECE1 WT group xenograft tumors (Fig. 7d-f & Fig. S7), which was consistent with our in vitro results. Considering that OS tends to metastasize, we injected 143B cells labeled with both a luminescent dye and GFP into the tail vein for seeding into the lung cavity. In vivo bioluminescence imaging demonstrated that CircECE1 WT promoted the metastasis of OS cells in situ, whereas CircECE1 MUT cells were unable to form lung metastases (Fig. 7g), which is similar to the results of the subcutaneous model. Further, we measured TXNIP and glycolytic enzyme expression in chondroma and OS patient samples using IHC and found decreased TXNIP expression and increased glycolytic enzyme expression in OS (Fig. 7h). Altogether, these findings indicate that CircECE1 activates c-Myc-mediated energy metabolism by preventing its degradation by SPOP in OS (Fig. 7i).

## Discussion

Preliminary studies have gradually illuminated the function and molecular mechanisms of circRNAs. circRNAs play a significant role in the development and prognosis of various cancers. Our previous study showed that *CircFAT1* can act as an endogenous competitive RNA to promote OS development through regulation of the Hippo signaling pathway^13^. *CircTADA2A* accelerates OS proliferation and metastasis^12^, and *circLARP4* acts as a sponge to regulate the expression of LARP4 by interacting with *miR-424-5p* in gastric cancer [27]. Furthermore, circRNAs are involved in regulating lung cancer phenotype by serving as endogenous competitive RNAs during carcinogenesis and proliferation [27, 28]. However, few current studies have examined the specific biological function and molecular mechanism of circRNAs in OS. circRNAs, which are evolutionarily conserved in mammals, possess multiple regulatory mechanisms owing to their specific covalently closed-loop construction. Therefore, more concrete studies are urgently needed to determine the functions and molecular mechanisms of circRNAs.

In this study, we found that *CircECE1* is upregulated in OS cells and OS tissues and interacts with c-Myc. We further explored the consequences of *CircECE1* upregulation. *CircECE1* promoted OS cell proliferation in vitro and growth in vivo. *CircECE1* promoted OS cell migration in vitro and colonization in the lung in vivo. Together, these data support our conclusion that *CircECE1* has pleiotropic effects on proliferation, tumor growth, invasion, colonization, and metastasis. Therefore, we conclude that *CircECE1* has oncogenic activity and promotes OS progression.

circRNAs function as signaling pathway regulators in gene expression and other important cellular processes [29, 30]. Many molecular functions are performed by circRNAs to implement their cellular effects, such as affecting gene cotranscription and binding to RNA or regulating protein translation by forming nuclear or cytoplasmic complexes [31–37]. Here, we demonstrated that *CircECE1* knockdown decreased the levels of c-Myc protein. We further demonstrated that c-Myc directly bound the *TXNIP* promoter to transcriptionally regulate *TXNIP* expression in OS cells. c-Myc is a crucial proto-oncogene in multiple malignant tumors and is closely associated with tumorigenesis, cell cycle regulation, and cell proliferation [38]. Moreover, c-Myc plays a critical role in invasion and metastasis [39]. Our previous studies showed that OS cells have higher c-Myc protein levels, indicating the presence of important c-Myc regulation mechanisms at the protein level^13^. Recently, Geng et al [40] found that the E3 ubiquitin ligase adapter SPOP can promote c-Myc degradation via ubiquitination. We found that the SPOP binding motif overlapped with the *circECE1* binding site. Therefore, we speculated that *CircECE1* may compete with SPOP to prevent SPOP-mediated degradation. Our results indicated that *CircECE1* interacts with c-Myc but not SPOP and acts to prevent SPOP-induced c-Myc degradation.

TXNIP is a glycolysis-related gene that we found to be regulated by *CircECE1.* We examined whether *CircECE1* altered glycolysis via TXNIP in OS. Previous studies have shown that TXNIP is a cancer suppressor gene in numerous solid tumors and hematological malignancies [41, 42]. Moreover, recent research revealed that TXNIP is a potent negative regulator of glucose uptake and aerobic glycolysis [43]. In our study, these results were confirmed by q-PCR and western blot analysis, which showed that both the mRNA and protein levels of TXNIP were remarkably higher in *CircECE1* knockdown OS cells than in control cells, indicating that TXNIP was regulated by *CircECE1*, predominantly via transcriptional regulation.

## Conclusions

Our research provides new insights into the role of circRNAs in OS and presents evidence for the molecular mechanisms by which circRNAs participate in the Warburg effect. This study identified *CircECE1* as a positive regulator of glucose metabolism and showed that it plays a crucial role in OS progression. Mechanistically, *CircECE1* interacts with c-Myc to prevent SPOP-induced c-Myc ubiquitination and degradation and then activates c-Myc-TXNIP signaling to regulate the Warburg effect. These results may help identify new diagnostic and therapeutic targets for OS.

## Supplementary Information

**Additional file 1: Supplementary Figure S1**. The circRNAs with high affinity to c-Myc. The ten circRNAs with high affinity to c-Myc were verified by an RIP assay. **Supplementary Figure S2**. The knockdown efficiency of CircECE1. A The expression levels of CircECE1 in 143B and U2OS cells after transfection of CircECE1 or control siRNAs were detected by real-time PCR. Data represents the mean ± SD (n = 3). * P < 0.05. Data represent the mean ± SD (n = 3). B The expression levels of ECE1 mRNA and CircECE1 in 143B and U2OS cells after stable transfection of CircECE1 short hairpin RNAs or vector plasmids were detected by real-time PCR. Data represents the mean ± SD (n = 3). * P < 0.05. C The expression levels of ECE1 mRNA and CircECE1 in 143B and U2OS cells after stable transfection of CircECE1 plasmids were detected by real-time PCR. Data represents the mean ± SD (n = 3). * P < 0.05. **Supplementary Figure S3**. The combination of CircECE1 and c-Myc. A Prediction of the binding position of CircECE1 to the c-Myc protein (catRAPID). B Prediction of the binding sequence of CircECE1 to the c-Myc protein (CISBP-RNA). C-D Schematic diagram of CircECE1 full-length and truncated fragments(C); The interaction of CircECE1 truncated fragments with c-Myc in 293T cells was verified by an RIP assay (D). E CircECE1 sequence labeling c-myc-binding site (red) and the mutated nucleotides (red). F The expression levels of CircECE1 in U2OS and 143B cells after stable transfection of CircECE1 WT/MUT or vector plasmids were detected by real-time PCR. Data represents the mean ± SD (n = 3). * P < 0.05. **Supplementary Figure S6**. The knockdown efficiency of c-Myc and the overexpression efficiency of TXNIP. A The expression levels of C-Myc in U2OS and 143B cells after transfection of c-Myc or control siRNAs were detected by real-time PCR. B The expression levels of TXNIP in U2OS and 143B TXNIP OE stable cells were detected by real-time PCR. Data represents the mean ± SD (n = 3). * P < 0.05. Data represent the mean ± SD (n = 3). **Supplementary Figure S7**. The overexpression efficiency of CircECE1 in vivo. The expression levels of CircECE1 in tumors formed by CircECE1 WT/MUT overexpression in osteosarcoma cells were detected by real-time PCR. Data represents the mean ± SD (n = 3). * P < 0.05.

## Data Availability

The datasets used and/or analysed during the current study are available from the corresponding author on reasonable request.

## Abbreviations

OS: Osteosarcoma
circRNAs: circular RNAs
*TXNIP*: Thioredoxin binding protein
HIF: Hypoxia-inducible factor
RIP: RNA immunoprecipitation
shRNA: Small hairpin RNA
FISH: RNA fluorescence in situ hybridization
SPOP: Speckle-type POZ
SBC: SPOP binding motif
qRT-PCR: Quantitative real-time polymerase chain reaction
