# Supplementary material for: CircECE1 activates energy metabolism in osteosarcoma by stabilizing c-Myc
Source: Mol Cancer. 2020 Oct 26;19:151. doi: 10.1186/s12943-020-01269-4 (PMC7586679; doi:10.1186/s12943-020-01269-4)
Supplement: Supplementary file 1 — Additional file 1: Supplementary Figure S1. The circRNAs with high affinity to c-Myc. The ten circRNAs with high affinity to c-Myc were verified by an RIP assay. Supplementary Figure S2. The knockdown efficiency of CircECE1. A The expression levels of CircECE1 in 143B and U2OS cells after transfection of CircECE1 or control siRNAs were detected by real-time PCR. Data represents the mean ± SD (n = 3). * P < 0.05. Data represent the mean ± SD (n = 3). B The expression levels of ECE1 mRNA and CircECE1 in 143B and U2OS cells after stable transfection of CircECE1 short hairpin RNAs or vector plasmids were detected by real-time PCR. Data represents the mean ± SD (n = 3). * P < 0.05. C The expression levels of ECE1 mRNA and CircECE1 in 143B and U2OS cells after stable transfection of CircECE1 plasmids were detected by real-time PCR. Data represents the mean ± SD (n = 3). * P < 0.05. Supplementary Figure S3. The combination of CircECE1 and c-Myc. A Prediction of the binding position of CircECE1 to the c-Myc protein (catRAPID). B Prediction of the binding sequence of CircECE1 to the c-Myc protein (CISBP-RNA). C-D Schematic diagram of CircECE1 full-length and truncated fragments(C); The interaction of CircECE1 truncated fragments with c-Myc in 293T cells was verified by an RIP assay (D). E CircECE1 sequence labeling c-myc-binding site (red) and the mutated nucleotides (red). F The expression levels of CircECE1 in U2OS and 143B cells after stable transfection of CircECE1 WT/MUT or vector plasmids were detected by real-time PCR. Data represents the mean ± SD (n = 3). * P < 0.05. Supplementary Figure S6. The knockdown efficiency of c-Myc and the overexpression efficiency of TXNIP. A The expression levels of C-Myc in U2OS and 143B cells after transfection of c-Myc or control siRNAs were detected by real-time PCR. B The expression levels of TXNIP in U2OS and 143B TXNIP OE stable cells were detected by real-time PCR. Data represents the mean ± SD (n = 3). * P < 0.05. Data represent [file 12943_2020_1269_MOESM1_ESM.docx]

­Supplementary Figures


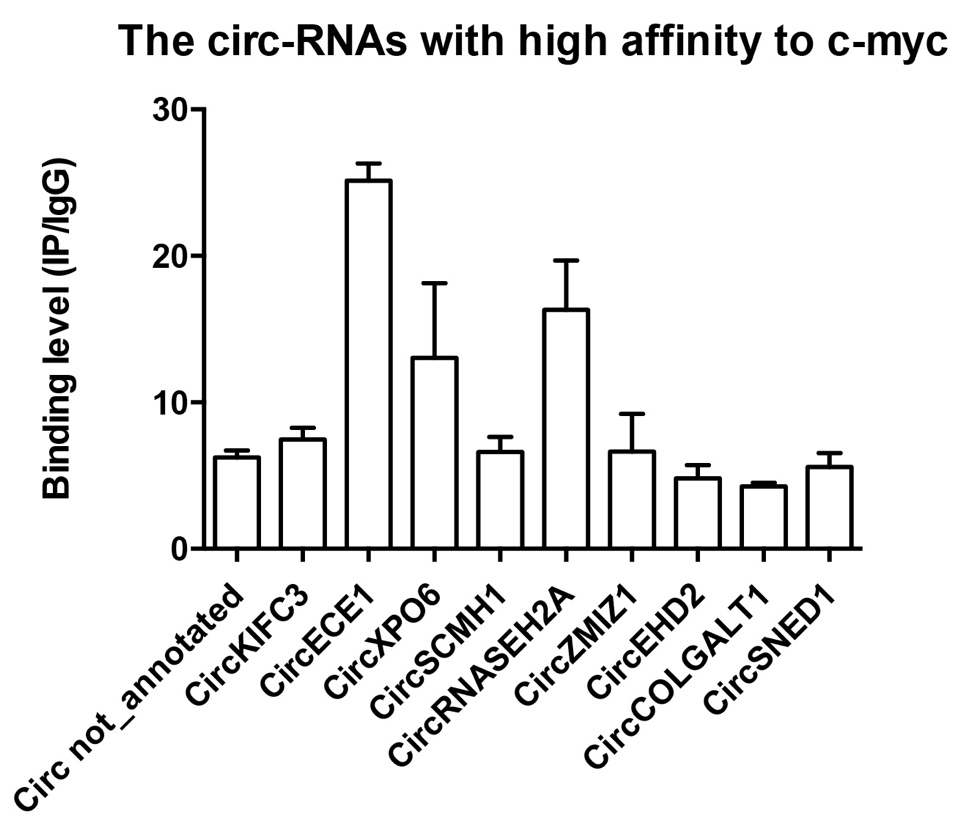


Supplementary FigureS1. The circRNAs with high affinity to c-Myc.

The ten circRNAs with high affinity to c-Myc were verified by an RIP assay.


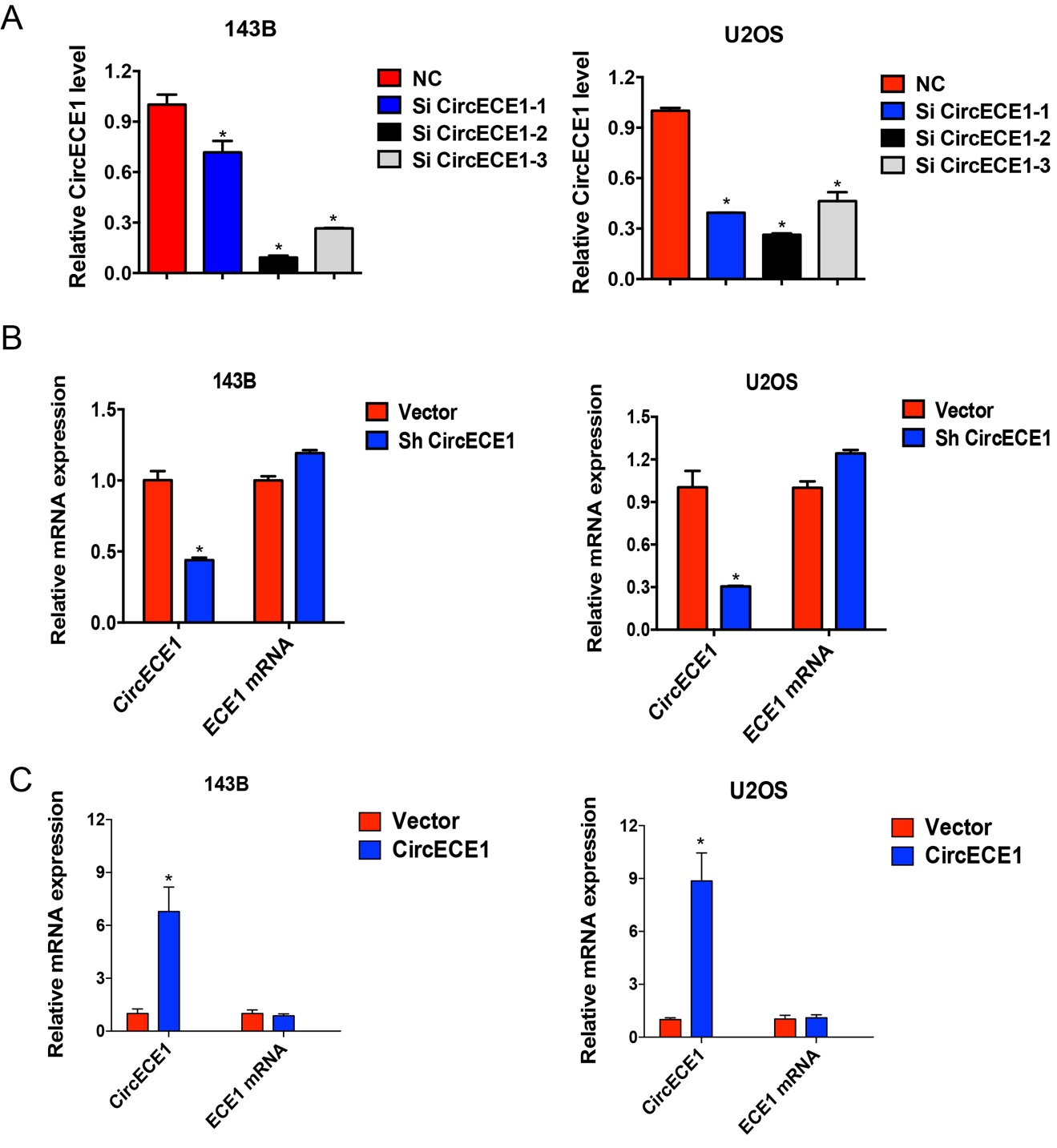


Supplementary Figure S2. The knockdown efficiency of CircECE1.

A The expression levels of CircECE1 in 143B and U2OS cells after transfection of CircECE1 or control siRNAs were detected by real-time PCR. Data represents the mean ± SD (n = 3). * P < 0.05. Data represent the mean ± SD (n = 3).

B The expression levels of ECE1 mRNA and CircECE1 in 143B and U2OS cells after stable transfection of CircECE1 short hairpin RNAs or vector plasmids were detected by real-time PCR. Data represents the mean ± SD (n = 3). * P < 0.05.

C The expression levels of ECE1 mRNA and CircECE1 in 143B and U2OS cells after stable transfection of CircECE1 plasmids were detected by real-time PCR. Data represents the mean ± SD (n = 3). * P < 0.05.


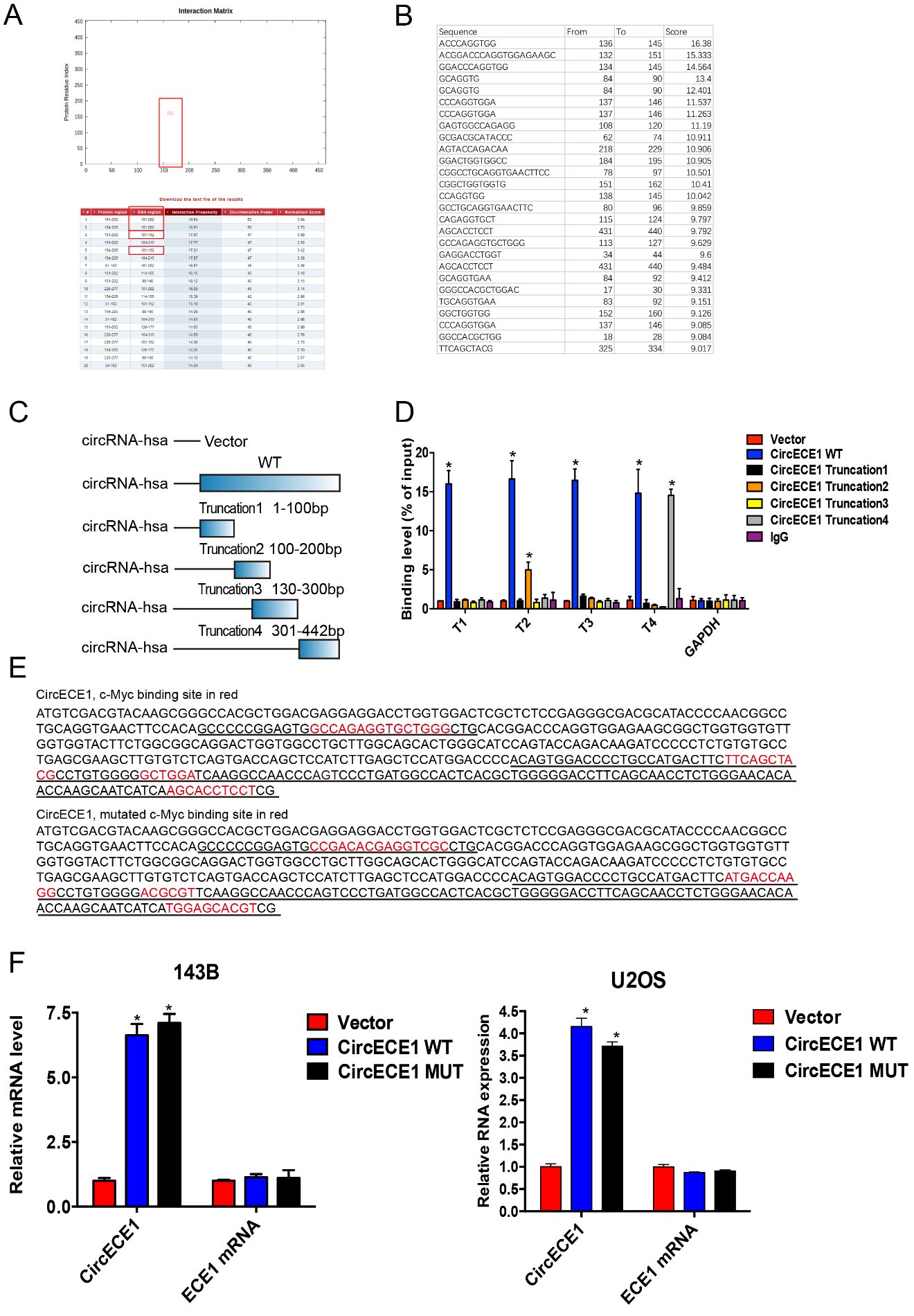


Supplementary Figure S3. The combination of CircECE1 and c-Myc.

A Prediction of the binding position of CircECE1 to the c-Myc protein (catRAPID).

B Prediction of the binding sequence of CircECE1 to the c-Myc protein (CISBP-RNA).

C-D Schematic diagram of CircECE1 full-length and truncated fragments(C); The interaction of CircECE1 truncated fragments with c-Myc in 293T cells was verified by an RIP assay (D).

E CircECE1 sequence labeling c-myc-binding site (red) and the mutated nucleotides (red).

F The expression levels of CircECE1 in U2OS and 143B cells after stable transfection of CircECE1 WT/MUT or vector plasmids were detected by real-time PCR. Data represents the mean ± SD (n = 3). * P < 0.05.


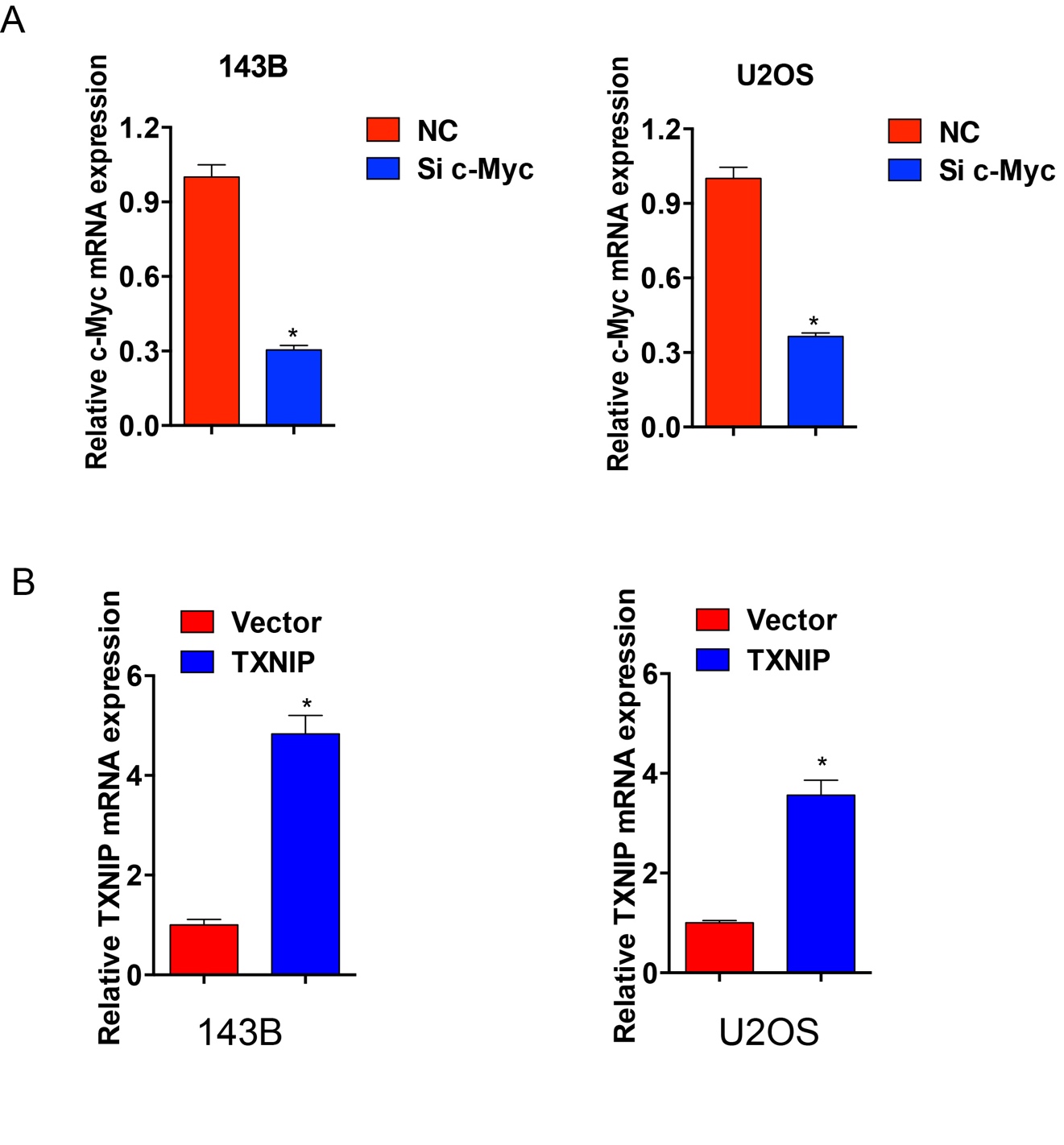


Supplementary Figure S6. The knockdown efficiency of c-Myc and the overexpression efficiency of TXNIP.

A The expression levels of C-Myc in U2OS and 143B cells after transfection of c-Myc or control siRNAs were detected by real-time PCR.

B The expression levels of TXNIP in U2OS and 143B TXNIP OE stable cells were detected by real-time PCR. Data represents the mean ± SD (n = 3). * P < 0.05. Data represent the mean ± SD (n = 3).


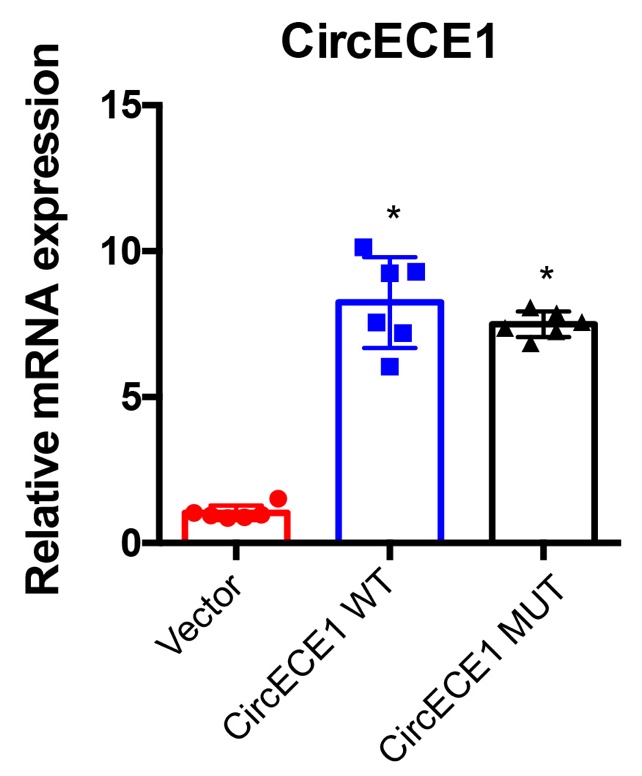


Supplementary Figure S7. The overexpression efficiency of CircECE1 in vivo.

The expression levels of CircECE1 in tumors formed by CircECE1 WT/MUT overexpression in osteosarcoma cells were detected by real-time PCR. Data represents the mean ± SD (n = 3). * P < 0.05.
